# Association between dietary diversity, sedentary time outside of work and depressive symptoms among knowledge workers: a multi-center cross-sectional study

**DOI:** 10.1186/s12889-023-17567-7

**Published:** 2024-01-02

**Authors:** Lijun Li, Pingting Yang, Yinglong Duan, Jianfei Xie, Min Liu, Yi Zhou, Xiaofei Luo, Chun Zhang, Ying Li, Jiangang Wang, Zhiheng Chen, Xiaohong Zhang, Andy S. K. Cheng

**Affiliations:** 1 Health Management Center, The Third Xiangya Hospital of Central South University, Changsha, China; 2 Nursing Department, The Third Xiangya Hospital of Central South University, Changsha, China; 3 Xiangya Nursing School of Central South University, Changsha, China; 4 Department of Rehabilitation Sciences, The Hong Kong Polytechnic University, Kowloon, Hong Kong

**Keywords:** Dietary diversity, Sedentary, Depressive symptoms, Knowledge workers, Cross sectional

## Abstract

**Background:**

Low-diversity diets and sedentary status are risk factors for depressive symptoms, while knowledge workers were ignored before. The purpose of this current study was to examine the relationship between dietary diversity, sedentary time spent outside of work, and depressive symptoms among knowledge workers.

**Study design and methods:**

This was a multicenter and cross‐sectional design that included 118,723 knowledge workers. Participants self-reported online between January 2018 and December 2020. Demographic information, the Dietary Diversity Scale, the Patient Health Questionnaire-9, dietary habits (which included eating three meals on time, midnight snacking, overeating, social engagement, coffee consumption, sugary drink consumption, smoking and alcohol use), sedentary time spent outside of work and physical activity were investigated.

**Results:**

The relationships between demographic information, dietary habits and dietary diversity, and depressive symptoms were estimated. Compared with the first and second levels of dietary diversity, the third level of dietary diversity (OR: 0.91; 95% CI: 0.84–0.98) reduced the risk of depressive symptoms. Knowledge workers with different degrees of sedentary status (2–4 h (OR: 1.11; 95% CI: 1.07–1.14), 4–6 h (OR: 1.21; 95% CI: 1.17–1.26), and > 6 h (OR: 1.49; 95% CI: 1.43–1.56), presented a progressively higher risk of depressive symptoms.

**Conclusion:**

High amounts of sedentary time spent after work and low levels of dietary diversity are risk factors for depressive symptoms. In addition, an irregular diet and overeating are also major risk factors for knowledge workers.

## Introduction

Knowledge workers are defined as people whose jobs consist primarily of creating and/or transforming knowledge into original products [1]. They implicitly select and weigh key factors in decision-making in their particular problem domain [2], and apply information and communication technology to exchange meaning [3]. This group is critical to currently service- and knowledge-based economy, in which innovation and creativity dominate [4]. With the rapid development of technology and society, the need for knowledge workers is increased [5]. However, because of the nature and characteristics of the work, this group of workers faces numerous health and psychological problems, such as depressive symptoms [1].

High levels of sedentary behavior have been identified as a risk factor for depressive symptoms and cardiometabolic health issues [6–8]. According to our knowledge, because of the need to complete certain tasks, sedentary time at work is not entirely under an employee's control. Therefore, health workers need to focus their intervention programs on reducing the amount of sedentary time spent outside of work hours. Research indicated that sedentary time and dietary patterns positively influenced the association between obesity and psychopathological symptoms in overweight/obese subjects [9]. In addition, there is also a significant association between sedentary and unhealthy dietary behaviors and psychological health-related outcomes in high school students [10]. Studies also suggested that depressive symptoms are associated with a sedentary lifestyle and dietary patterns in different groups, such as medical students, women, and older adults [11–13].

Dietary diversity has been considered an essential component of a healthy diet [14], a key indicator of high dietary quality in different populations [15, 16]. Dietary diversity score (DDS) is an a priori-defined dietary quality evaluation index[17]. United States Department of Agriculture’s (USDA) Dietary Guidelines and Food Guide Pyramid states that dietary diversity is one of the characteristics of a healthy diet [18]. The Chinese Dietary Guideline also indicates that people should consume as many kinds of food as possible [19]. Undernutrition and low-diversity diets may be risk factors for the occurrence or progression of mental disorders [20]. Globally, approximately 5% of excess mortality can be attributed to inadequate consumption of fruits and vegetables [21]. Consuming diverse vegetables contribute to reducing depressive symptoms [22]. diversity diet is a significant mediator between dietary quality and depression [23]. Balanced diet is important for people’s health, previous studies indicated that the low vitamin D levels are associated with a variety of diseases [24], different vitamin supplement usage may associate with the disease management [25, 26].

Knowledge workers need to continuously learn and compete to avoid becoming obsolete. As a result, they are at high risk for depression under chronic high stress [27]. Low-diversity diets and sedentary status are risk factors for depressive symptoms. The association between dietary diversity, sedentary and depressive symptoms was investigated in different populations, especially for adolescent. One study found that depressive symptoms in overweight and obese women from disadvantaged communities are associated with dietary intake but not physical activity [28]. A study found a connection between sedentary and dietary behaviors and depressive symptoms in adolescents [29]. Increased unhealthy dietary pattern scores and physical activity guideline attainment were associated with depressive symptoms in males, whereas screen time guideline attainment (sedentary) was associated with depressive symptoms in females [29].

However, the above studies were not performed using a large population, nor did the study groups include knowledge workers [30–32]. There rarely studies have examined the association between dietary diversity or sedentary behavior outside of work and depressive symptoms among knowledge workers. Therefore, the purpose of this study was to (1) determine the dietary diversity score, sedentary time spent outside of work, and depression among a large population in China; (2) determine the association between the dietary diversity score and sedentary time spent outside of work and depression; and (3) detect other influencing factors of depression in this group for further systematic review.

## Methods

### Participants

The participants were recruited from eight health management centers of general tertiary hospitals located in eastern, southern, central and northern China, with convenience sampling to obtain the sample. Inclusion criteria were recruited at the study sites: (1) knowledge workers who process information to develop knowledge and to generate theories and concepts in the workplace, defined by Peter Drucker [33]; (2) age 18 years old or above; (3) could read and understand the questionnaire items in Chinese; (4) completed all general information, lifestyle and dietary surveys, and depressive symptom surveys and (5) provided informed consent. Exclusion criteria were questionnaires that did not answer key questions, questionnaires with contradictory answers to evaluation questions, and questionnaires that showed an overly regular pattern of responses.

### Study design and procedures

**Fig. 1:**
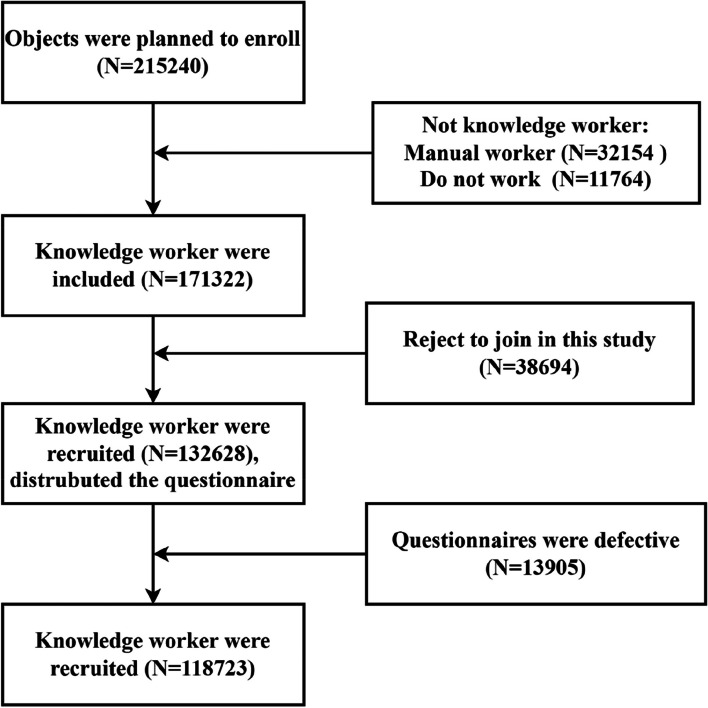
The flowchart of this study

This was a multicenter, cross-sectional study conducted between January 2018 and December 2020. Before the routine screening, participants completed an online survey at the medical examination center through the website, including 86 items (https://new.selfhealth.com.cn/#/login). Participants completed a detailed personal questionnaire containing demographic characteristics and dietary and mental health information. This study was approved by the Research and Development of Decision Support System for Residents' Healthy Lifestyle Self-reported Health Guidance Technology Intervention. Each agency involved in the survey received agency approval. Participation in the study was entirely voluntary, and there was no reward for participants. The IP addresses of participants were recorded to prevent double voting. The flowchart of this study was shown in Fig. 1.

### Measures

#### Demographics and clinical characteristics

We collected the following demographic data from each participant: gender, age, marital status, and BMI degrees, which were categorized as underweight, normal, overweight, and obese for BMI < 18.5 kg/m^2^, 18.5–24.9 kg/m^2^, 25–27.9 kg/m^2^, and ≥ 28 kg/m^2^, respectively [34].

#### Physical activity and Sedentary time outside of work

Physical activity refers to exercising during non-working time, assessed by whether or not an individual engages in physical activity. Except for working sitting, working adults spent 0.97 to 2.19 h in different affairs to the sedentary outside of work [35]. In this study, sedentary outside of work is set to a level for every 2 h, divided into four degrees: < 2 h, 2–4 h, 4–6 h, and > 6 h in one day.

#### Dietary Diversity Scale, DDS

The DDS is based on the Chinese Food Pagoda [36], which classifies foods into nine categories: grains (including cereals, roots and potatoes), vegetables, fruits, livestock meat, fish and shrimp, eggs, milk and dairy products, beans or soy products, and oils or fats. Each category of food consumed is scored as one point, with a maximum of nine points, regardless of the frequency and quantity of the food consumed. It was based on the participant's recall of the food eaten in the past three days. If the participant consumed any of the above foods, he would receive one point in that food category; otherwise, he would score zero points. Consumption of different foods in the same category is not double-counted. The total scoring is divided as follows: 1–5 is insufficient [DDS-1], 5–7 is moderate [DDS-2], and 8–9 is adequate [DDS-3] [37]. The DDS was widely used in children and adults [38].

#### Dietary habits

In this section, we investigate the eating habits of the participants, including eating three meals on time, midnight snacks, overeating, social engagement, coffee consumption, sugary drink consumption, smoking and alcohol use.

Questionnaires included a *Yes/Basically/No* selection for eating three meals on time, which means whether the diet is regular or not; a *No/Occasionally/Often* selection for midnight snacks; a *No or 1–2 times per month/1–2 times per week/* > *3 times per week* selection for social engagement; a *No/Moderate/Excessive* selection for alcohol consumption (Dietary Guidelines for Chinese Residents suggest that for men, moderate drinking is defined as no more than 25 g of alcohol a day; for women, moderate alcohol consumption is 15 g per day); a *No/Yes* selection for overeating, coffee consumption, sugary drink consumption, and smoking.

#### Patient Health Questionnaire-9 (PHQ-9)

The PHQ-9, which includes 9 items, was applied to evaluate depressive symptoms. The scores range from 0 to 27, and the higher the score was, the more severe the depressive symptoms were. A total score ≥ 10 indicates clinically relevant depressive symptoms. Otherwise, it indicated participants without depressive symptoms [39, 40]. The Cronbach'α coefficient of the PHQ-9 is 0.89.

### Ethical considerations

The study was conducted according to the guidelines of the Declaration of Helsinki, and ethical approval was granted by the Ethics Committee of The Third Xiangya Hospital of Central South University (No. 2020-S587). All participants have signed the consent form.

### Statistical analysis

We used SPSS 26.0 software for Windows (IBM Corp, Armonk, New York) to analyze the data. All variables were categorical data, described by number and percentage. We performed the χ^2^ test to compare each level of dietary diversity and with/without depressive symptoms. To adjust for confounding factors, multilevel binary logistic regression was used to assess the relationship between demographic characteristics, physical activity, sedentary, DDS, and dietary habits. The correlation between these variables also is presented by the Spearman correlation coefficient. Model 1: adjusted for age, gender, marriage, and BMI; Model 2: further adjusted for physical activity and sedentary; Model 3: further adjusted for dietary habits and DDS. The indicator for all independent variables was followed first. A p-value less than 0.05 is typically considered to be statistically significant.

## Results

### Demographic characteristics

We collected all data from 118,723 knowledge workers. A total of 65.6% of them were male, and most were 25 to 44 years old (55.0%) and married (87.1%), the percentages of participants aged 45–64, 65 and older, 24 and younger were 38.5%, 3.7%, and 2.8% respectively. Approximately half (49.0%) of the respondents’ BMIs were within the normal range (18.5–23.9 kg/m^2^), while 38.4% were overweight (24–27.9 kg/m^2^). Nearly one-third of the respondents never physical activity. 33.5% were sedentary outside of work for more than 4 h. Regarding dietary habits, 69.0% ate three meals on time, most of the respondents (93.6%) did not overeat, and approximately three-quarters of the respondents never or only rarely (1–2 times a month) attended a social engagement. More than half of the respondents never drank coffee (67.3%), consumed alcohol (55.6%), smoked (52.0%), or consumed sugary drinks (51.5%). Table 1 presents the details of the demographic information.

**Table 1:** Demographic information, sedentary, eating habits, and the effect on dietary diversity and depression (*N*=118723)

Variables	N (%)	Dietary Diversity			χ^2^	*P*	Depressive symptoms		χ^2^	*P*
		1-5 (3.0%)	6-7 (25.1%)	8-9 (71.9%)			No (68.5%)	Yes (31.5%)		
Gender					266.18	<0.001			264.79	<0.001
Male	77838 (65.6)	2105 (2.7)	18596 (23.9)	57137 (73.4)			54548 (70.1)	23290 (29.9)		
Female	40885 (34.4)	1430 (3.5)	112337 (27.5)	28218 (69.0)			26764 (65.5)	14121 (34.5)		
Age, y					58.03	<0.001			1271.99	<0.001
≤24	3220 (2.8)	114 (3.5)	904 (28.1)	2202 (68.4)			1922 (59.7)	1298 (40.3)		
25-44	65306 (55.0)	2037 (3.1)	16605 (25.4)	46664 (71.5)			42232 (64.7)	23074 (35.3)		
45-64	45717 (38.5)	1253 (2.7)	11140 (24.4)	33324 (72.9)			33800 (73.9)	11917 (26.1)		
≥65	4480 (3.7)	131 (2.9)	1184 (26.4)	3165 (70.6)			3358 (75.0)	1122 (26.1)		
Marriage					64.72	<0.001			428.29	<0.001
Unmarried	13211 (11.1)	510 (3.9)	3503 (26.5)	9198 (69.6)			8021 (60.7)	5190 (39.3)		
Married	103416 (87.1)	2973 (2.9)	25773 (24.9)	74670 (72.2)			71908 (69.5)	31508 (30.5)		
Divorced or widowed	2096 (1.8)	52 (2.5)	557 (26.6)	1487 (70.9)			1383 (66.0)	713 (34.0)		
BMI, kg/m^2^					192.55	<0.001			237.03	<0.001
< 18.5	4342 (3.6)	198 (4.6)	1292 (29.8)	2852 (65.7)			2716 (62.6)	1625 (37.4)		
18.5-23.9	58134 (49.0)	1856 (3.2)	15034 (25.9)	41244 (70.9)			38943 (67.0)	19191 (33.0)		
24-27.9	45580 (38.4)	1198 (2.6)	10960 (24.0)	33422 (73.3)			32111 (70.4)	13469 (29.6)		
≥28	10667 (9.0)	283 (2.7)	2547 (23.9)	7837 (73.5)			7542 (70.7)	3125 (29.3)		
Physical activity					2502.53	<0.001			1715.03	<0.001
None	41114 (34.6)	2032 (4.9)	13006 (31.6)	26076 (63.4)			25004 (60.8)	16110 (39.2)		
Yes	77609 (65.4)	1503 (1.9)	16827 (21.7)	59279 (76.4)			56308 (72.6)	21301 (27.4)		
Sedentary					381.60	<0.001			1089.56	<0.001
< 2 hours	29118 (24.5)	995 (3.4)	7450 (25.6)	20673 (71.0)			21307 (73.2)	7811 (26.8)		
2-4 hours	49809 (42.0)	1183 (2.4)	11623 (23.3)	37003 (74.3)			34836 (69.9)	14973 (30.1)		
4-6 hours	24125 (20.3)	765 (3.2)	6192 (25.7)	17168 (71.2)			15956 (66.1)	8169 (33.9)		
>6 hours	15671 (13.2)	592 (3.8)	4568 (29.1)	10511 (67.1)			9213 (58.8)	6458 (41.2)		
Eating three meals on time					876.75	<0.001			3531.03	<0.001
Yes	81968 (69.0)	2187 (2.7)	19500 (23.8)	60281 (73.5)			60412 (73.7)	21556 (26.3)		
Basically	31116 (26.2)	970 (3.1)	8284 (26.6)	21862 (70.3)			18144 (58.3)	12972 (41.7)		
No	5639 (4.8)	378 (6.7)	2049 (36.3)	3212 (57.0)			2756 (48.9)	2883 (51.1)		
Midnight snacks					239.05	<0.001			2136.64	<0.001
No	69011 (58.1)	2153 (3.1)	17539 (25.4)	49319 (71.5)			50821 (73.6)	18190 (26.4)		
Occasionally	45744 (38.5)	1186 (2.6)	11017 (24.1)	33541 (73.3)			28350 (62.0)	17394 (38.0)		
Often	3968 (3.4)	196 (4.9)	1277 (32.2)	2495 (62.9)			2141 (54.0)	1827 (46.0)		
Overeating					325.79	<0.001			1210.80	<0.001
Yes	7627 (6.4)	449 (5.9)	2199 (28.8)	4979 (65.3)			3858 (50.6)	3769 (49.4)		
No	111096 (93.6)	3086 (2.8)	27634 (24.9)	80376 (72.3)			77454 (69.7)	33642 (30.3)		
Social engagement					119.17	<0.001			199.97	<0.001
No or 1-2 times/month	87383 (73.6)	2757 (3.2)	22388 (25.6)	62238 (71.2)			60796 (69.6)	26587 (30.4)		
1-2 times/week	26110 (22.0)	613 (2.3)	6079 (23.3)	19418 (74.4)			17227 (66.0)	8883 (34.0)		
Timesweek	5230 (4.4)	165 (3.2)	1366 (26.1)	3699 (70.7)			3289 (62.9)	1941 (37.1)		
Coffee					1049.08	<0.001			133.06	<0.001
No	79946 (67.3)	2872 (3.6)	21844 (27.3)	55230 (69.1)			55620 (69.6)	24326 (30.4)		
Yes	38777 (32.7)	663 (1.7)	7989 (20.6)	30125 (77.7)			25692 (66.3)	13085 (33.7)		
Sugary drinks					504.57	<0.001			1564.19	<0.001
No	57522 (48.5)	2127 (3.7)	15643 (27.2)	39752 (69.1)			42560 (74.0)	14962 (26.0)		
Yes	61201 (51.5)	1405 (2.3)	14190 (23.2)	45603 (74.5)			38752 (63.3)	22449 (36.0)		
Smoking					25.46	<0.001			8.94	0.003
No	61743 (52.0)	1940 (3.1)	15848 (25.7)	43955 (71.2)			39264 (68.9)	17716 (31.1)		
Yes	56980 (48.0)	1595 (2.8)	13985 (24.5)	41400 (72.7)			42048 (68.1)	19695 (31.9)		
Alcohol					448.78	<0.001			31.403	<0.001
No	66067 (55.6)	2342 (3.5)	17742 (26.9)	45983 (69.6)			44814 (67.8)	21253 (32.2)		
Moderate	35450 (29.9)	781 (2.2)	8110 (22.9)	26559 (74.9)			24634 (69.5)	10816 (30.5)		
Excessive	17206 (14.5)	412 (2.4)	3981 (23.1)	12813 (74.5)			11864 (69.0)	5342 (31.0)		

### The dietary diversity of participants

As shown in Table 1, the percentages for the dietary diversity levels of 9 and 8 were 37.6% and 34.3%, respectively. A total of 71.9% of the population achieved level 3 dietary diversity (*p* < 0.001). The percentage of individuals with a DDS3 was lower among those who did not eat three meals on time (χ2 = 876.75, *p* < 0.001) and among those who frequently ate midnight snacks (χ2 = 239.05, *p* < 0.001). In addition, the mean score was 7.98 ± 1.11 for all participants.

### The association between dietary diversity, sedentary time, and other variables and depressive symptoms

**Fig. 2:**
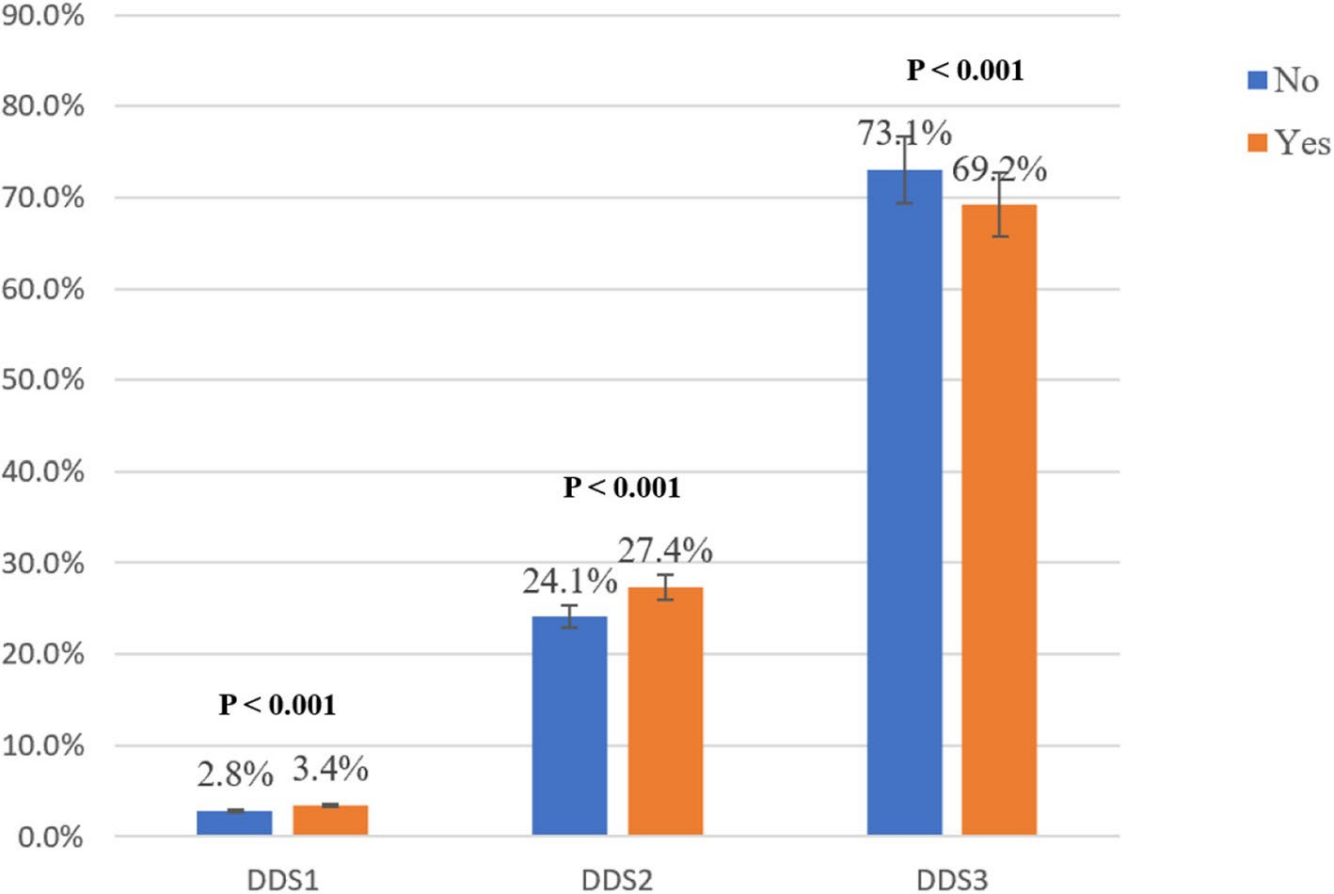
The depressive symptom distribution on different DDS groups. Abbreviations: DDS, Dietary Diversity Scale. Note: The data are presented by percentage of depressed and non-depressed participants in different DDS levels

**Fig. 3:**
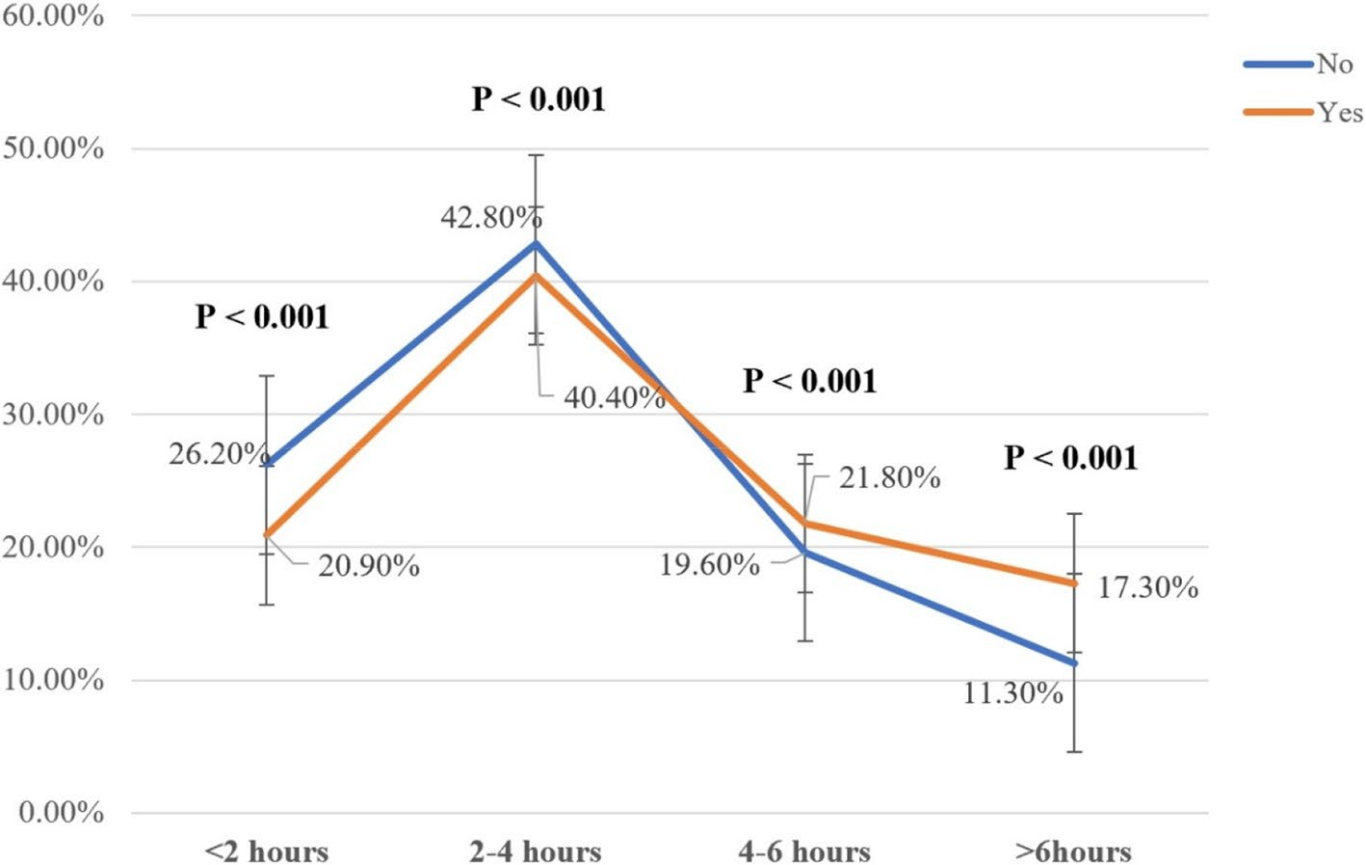
The influence of different sedentary time outside of work on depressive symptom. Note: The data are presented by percentage of depressed and non-depressed participants in different sedentary time outside of work

Among the study sample, 31.5% of the participants reported depressive symptoms. Figure 2 shows that a higher proportion of depressed than non-depressed participants had diets rated at the DDS-1 and DDS-2 levels, while a higher proportion of non-depressed participants had diets rated at a DDS-3 level (*p* < 0.001). When sedentary time reaches 4–6 h and above 6 h, the percentage of depression is higher than that of non-depressed people (*p* < 0.001) (Fig. 3).

Regarding eating habits, a higher percentage of depressive symptoms was significantly associated with not eating three meals on time, often having midnight snacks, and sugary drink consumption (*p* < 0.001) (Table 1). The younger the age of the participants was, the greater the proportion of depressive symptoms (*p* < 0.001). People who never physical activity had a higher rate of depressive symptoms (*p* < 0.001).

### Correlation between depressive symptoms, physical activity, sedentary, and dietary factors

The Spearman correlation analysis showed that depressive symptoms were associated with physical activity, sedentary, DDS, and all dietary habits variables except for alcohol (*p* < 0.01). DDS also correlated to all those variables without midnight snacks (*p* < 0.001) (Table 2).

**Table 2:** The correlations between depressive symptoms, physical activity, sedentary behaviors, and dietary factors in knowledge workers (*N*=118723)

Variables	(1)	(2)	(3)	(4)	(5)	(6)	(7)	(8)	(9)	(10)	(11)	(12)
(1) Depression	1.000											
(2) Physical activity	-0.120^c^	1.000										
(3) Sedentary	0.090^c^	-0.133^c^	1.000									
(4) DDS	-0.041^c^	0.141^c^	-0.022^c^	1.000								
(5) Eating three meals on time	0.172^c^	-0.178^c^	0.118^c^	-0.063^c^	1.000							
(6) Midnight snacks	0.134^c^	-0.156^c^	0.110^c^	0.006	0.279^c^	1.000						
(7) Overeating	-0.101^c^	0.083^c^	-0.075^c^	0.042^c^	-0.148^c^	-0.136^c^	1.000					
(8) Social engagement	0.040^c^	-0.018^c^	0.067^c^	0.024^c^	0.149^c^	0.185^c^	-0.175^c^	1.000				
(9) Coffee	0.033^c^	0.015^c^	0.026^c^	0.092^c^	0.086^c^	0.109^c^	-0.026^c^	0.058^c^	1.000			
(10) Sugary drinks	0.115^c^	-0.109^c^	0.088^c^	0.062^c^	0.185^c^	0.290^c^	-0.068^c^	0.052^c^	0.253^c^	1.000		
(11) Smoking	-0.009^b^	0.018^c^	-0.005	0.017^c^	-0.013^c^	-0.007^a^	0.019^c^	-0.021^c^	0.012^c^	0.017^c^	1.000	
(12) Alcohol	-0.014	0.075	0.017^c^	0.056^c^	0.042^c^	0.110^c^	-0.078^c^	0.339^c^	0.070^c^	0.026^c^	0.028^c^	1.000

*Abbreviations*: *DDS* Dietary Diversity Scale

^a^*p*< 0.05; ^b^*p*< 0.01; ^c^*p*< 0.001

### Multilevel logistic regression analysis of depressive symptoms

Table 3 shows the factors associated with multiple symptoms that were analyzed. Binary logistic regression models suggested that females [OR (Odds Ratio): 1.17; 95% CI (Confidence Interval): 1.13–1.20] reported an increased risk of depressive symptoms compared to males. The age groups of 45–64 years old (OR: 0.82; 95% CI: 0.76–0.90) and 65 years old and above (OR: 0.79; 95% CI: 0.71–0.88), those who were married (OR: 0.94; 95% CI: 0.90–0.98), those with the highest BMI level (OR: 0.88; 95% CI: 0.81–0.95), and those who physical activity (OR: 0.76; 95% CI: 0.74–0.78) had a reduced risk of depressive symptoms. Knowledge workers with different degrees of sedentary time: 2–4 h (OR: 1.11; 95% CI: 1.07–1.14), 4–6 h (OR: 1.21; 95% CI: 1.17–1.26), or > 6 h (OR: 1.49; 95% CI: 1.43–1.56); those who basically (OR: 1.61; 95% CI: 1.57–1.67) and never (OR: 1.89; 95% CI: 1.78–2.00) ate three meals on time; and those who partook in social engagements 1–2 times per week (OR: 1.04; 95% CI: 1.01–1.08) or more than three times per week (OR: 1.09; 95% CI: 1.03–1.17) all presented a progressively higher risk of depressive symptoms. Participants without the habit of overeating (OR: 0.59; 95% CI: 0.56–0.62), those who smoked (OR: 0.94; 95% CI: 0.91–0.96), those who reported moderate (OR: 0.95; 95% CI: 0.92–0.98) or excessive (OR: 0.94; 95% CI: 0.90–0.98) alcohol consumption, and those who reported DDS3 diets (OR: 0.91; 95% CI: 0.84–0.98) had a reduced risk of depressive symptoms. Conversely, consuming sugary drinks (OR: 1.30; 95% CI: 1.27–1.34) increased the risk of depressive symptoms.

**Table 3:** Adjusted associations between dietary and depressive symptoms after adjustment for other factors (*N*=118723)

**Variables**	**Model 1**	**Model 2**	**Model 3**
	**OR [95% CI]**		
**Gender**			
Male	1.00	1.00	1.00
Female	1.13 [1.10-1.16]^c^	1.11 [1.08-1.14]^c^	1.17 [1.13-1.20]^c^
**Age, y**			
≤24	1.00	1.00	1.00
45-64	0.66 [0.60-0.71]^c^	0.69 [0.64-0.76]^c^	0.82 [0.75-0.90]^c^
≥65	0.61 [0.55-0.68]^c^	0.66 [0.59-0.73]^c^	0.79 [0.71-0.88]^c^
**Marriage**			
Unmarried	1.00	1.00	1.00
Married	0.83 [0.80-0.87]^b^	0.84 [0.81-0.88]^b^	0.94 [0.90-0.98]^b^
**BMI, kg/m**^**2**^			
< 18.5	1.00	1.00	1.00
24-27.9	0.90 [0.84-0.97]^b^	0.94 [0.87-1.00]	0.93 [0.87-1.00]
≥28	0.89 [0.82-0.96]^b^	0.90 [0.83-0.97]^b^	0.88 [0.81-0.95]^b^
**Physical activity**			
None		1.00	1.00
Yes		0.65 [0.63-0.67]^c^	0.76 [0.74-0.78]^c^
**Sedentary**			
< 2 hours		1.00	1.00
2-4 hours		1.16 [1.12-1.19]^c^	1.11 [1.07-1.14]^c^
4-6 hours		1.34 [1.29-1.39]^c^	1.21 [1.17-1.26]^c^
> 6 hours		1.72 [1.65-1.79]^c^	1.49 [1.43-1.56]^c^
**Eating three meals on time**			
Yes			1.00
Basically			1.61 [1.57-1.67]^c^
No			1.89 [1.78-2.00]^c^
**Midnight snack**			
No			1.00
Occasionally			1.26 [1.23-1.30] ^c^
Often			1.21 [1.13-1.30] ^c^
**Overeating**			
Yes			1.00
No			0.59 [0.56-0.62]^c^
**Social engagement**			
No or 1-2 times/month			1.00
1-2 times/week			1.04 [1.01-1.08]^a^
timesweek			1.09 [1.03-1.17]^b^
**Sugary drinks**			
No			1.00
Yes			1.30 [1.27-1.34]^c^
**Smoking**			
No			1.00
Yes			0.94 [0.91-0.96]^c^
**Alcohol**			
No			1.00
Moderate			0.95 [0.92-0.98]^b^
Excessive			0.94 [0.90-0.98]^b^
**DDS**			
1-5			1.00
8-9			0.91 [0.84-0.98]^b^

Model 1: adjusted for age, gender, marriage, and BMI; Model 2: further adjusted for physical activity and sedentary; Model 3: further adjusted for dietary habits and DDS

*Abbreviations*: *CI* confidence interval, *DDS* Dietary Diversity Scale, *OR* odds ratio;

^a^*p*< 0.05; ^b^*p*< 0.01; ^c^*p*< 0.001

## Discussion

The study provides preliminary evidence of Chinese knowledge workers’ dietary diversity, sedentary time spent outside of work, depressive symptoms. We also explored the association between dietary diversity or sedentary time spent outside of work and depressive symptoms. In addition, gender, age, marital status, BMI, physical activity, eating habits and their association with depressive symptoms were investigated among knowledge workers. We found that a lower DDS score and greater sedentary time spent outside of work were significantly related to knowledge workers’ depressive symptoms. It was also surprising that 31.5% of participants reported depressive symptoms, which was marginally lower than the results reported in previous studies in Tehran (62.1%) and Korea (38.3%) [41, 42]. However, the sample sizes of those studies were smaller and limited to office workers. While 31.5% may not be a large percentage, because of the large sample size of this study, this population is too large to be ignored.

We found among those reporting diets in the DDS-1 and DDS-2 groups, the percentage of depressive symptoms was higher than in the non-depressed group, while the DDS-3 was inverse. An increase in dietary variety may reverse the percentage of depressive symptoms among knowledge workers. Besides, the DDS-3 degree is a protection factor against depressive symptoms in knowledge workers, and DDS is a negative association with depressive symptoms, which are similar to previous studies [43, 44]. Low DDS is a risk factor for depressive symptoms [45] and is inversely correlated with depressive symptoms [43]. The relationship between depression symptoms and single nutrients and foods, such as vitamins, folic acid and fish, is inconsistent [46]. Many nutrients are highly interrelated, and some may also influence the intestinal absorption of other nutrients [47]. A longitudinal study reported positive effects of greater food group diversity and maternal depressive symptoms [48]. Among adolescents, the proportion of moderate to severe and major depressive symptoms increased slightly as the DDS score decreased [49]. Dietary diversity in general, and diversity in certain food groups in particular, can reduce the risk of metabolism-related outcomes by improving the diversity of the gut microbiome [50]. which subsequently improves health outcomes and immune function[51]. The results of this study affirmed the above studies. However, most of the previous studies mainly concentrated on women or adolescents. Knowledge workers were ignored, which indicates that further research is needed among this group.

Regarding eating habits, knowledge workers who did not eat three meals regularly had an increased risk of depressive symptoms compared to those who ate three meals regularly. A regular diet is a protective factor against chronic stress, and most people with irregular diets have inadequate nutritional intake, especially folic acid, zinc and magnesium intake, which are beneficial for maintaining neurological function [52]. In addition, the hypothalamus is the main central structure regulating appetite and emotional responses; it is involved in the regulation of glucose metabolism while also regulating stress responses [53]. Therefore, dietary factors are closely related to the occurrence of depressive symptoms. Compared with those who overate, knowledge workers who never overate showed a lower risk of depressive symptoms. Hanna also indicated that emotional overeating was significantly associated with eating disorder characteristics and depressive symptoms [54].

Consuming sugary drinks daily was a predictor of having higher depressive symptomatology [55]. Sugar has been shown to have a very negative effect on brain proteins, mainly on neutrophic proteins, which are known to play a role in depressive symptoms because they protect the brain from oxidative stress and promote the growth of new brain cells [56]. Knowledge workers who smoked and consumed alcohol had a lower risk of depressive symptoms in our study. Lee et al. investigated the triple trajectories of alcohol use, tobacco use, and depressive symptoms among adults [57]. Two trajectories were presented: (1) low tobacco use, moderate alcohol use, and low depressive symptoms and (2) low alcohol use, no tobacco use, and high depressive symptoms. Moderate smoking and alcohol consumption may at some point alleviate depressive symptoms among knowledge workers.

Reducing sedentary time and increasing physical activity may ease depressive symptoms in different populations ranging from adolescents to middle-aged women to older adults [58–60]. Our study also showed similar results: the percentage of depressive symptoms was higher than non-depressive symptoms with the gradual increase in sedentary time spent outside of work. Physical activity can reduce the risk of depressive symptoms among knowledge workers. Transient mood disorders caused by sedentary activity may interact with acute stress to strengthen the pro-inflammatory response [61]. Meanwhile, Passive sedentary activity replaces physical activity and may result in social isolation, which is associated with depression [62]. Sedentary behavior is one of the main causes of depressive symptoms in elderly patients with hypertension [63]. Meanwhile, Raudsepp & Vink suggested that early depressive symptoms also predict later sedentary behavior [64]. Knowledge workers spend most of their workday sitting; they sit up to 82% of their work time [65]. Accordingly, this increasingly sedentary behavior has become a major public health risk [66]. Previous studies have explored the factors influencing and barriers to changing the amount of sedentary time knowledge workers engaged in each day [30, 31]. Moreover, numerous intervention programs and treatments have attempted to reduce sedentary behavior and increase physical activity levels among knowledge workers [32, 67, 68]. A 22-month period longitudinal study reported that an increase in sedentary behaviors was inversely associated with a change in physical activity [69]. Physical activity intervention also reduced total sedentary time[70]. We particularly investigated the amount of sedentary time spent outside of work rather than sedentary time spent during the workday among knowledge workers. As we understood, this study is the first to explore the amount of sedentary time spent outside of work in a large population of knowledge workers. Programs and mechanisms targeting sedentary time spent outside of work merit further research.

Furthermore, compared with males, females knowledge workers showed a higher risk of depressive symptoms, which is similar to China Mental Health Survey [71], indicated that female patients accounted for more than 60% of the total number of depression patients. In addition, female patients also have a higher willingness to share and actively seek treatment through friends and relatives, patient communities, and various social channels than male patients [71]. It is possible that even though there were fewer female participants in this study, more women than men reported depressive symptoms. Older participants had a lower risk of depressive symptoms in this study, and Ogasawara et al. also indicated a significant relationship between depressive symptoms onset and younger age [72]. In this study, marriage was a protective factor for depressive symptoms. The contribution of marriage to mental health was greatest if both married partners were employed [73].

### Strengths and limitations

This study with a large sample size to evaluate the dietary diversity in knowledge workers in China for the first time. Because the responses to this study were online and voluntary, the data are likely to come from people who had a higher chance of being depressed or of suffering from more severe depressive symptoms. This may be because those who are depressed or worried about depressive symptoms are more likely to voluntarily report their symptoms or answer a screening questionnaire. Additionally, this study was a cross-sectional design, and longitudinal changes in depressive symptoms among participants were not tracked. Meanwhile, the results may cause reverse causation because of the cross-sectional nature of the study.

Besides, the classification of physical activity is not sufficiently detailed. Another major limitation of this study is the use of the PHQ-9 to provide a measure of depressive symptoms and severity; the PHQ-9 is a self-report measure that, although well-established clinically, may not provide an accurate measure of specific symptoms. As a screening tool, the PHQ-9 provides a good starting point for identifying new measures of depressive symptoms, but more accurate and objective measures are needed in future studies. PHQ-9, like many other screening methods, is not biologically specific and does not provide information on the pathology of depressive symptoms or its progression. Future studies could improve the objectivity of the study with a case or physiological indicators, such as links to neurobiological function or cortisol levels [74, 75].

Despite these disadvantages, our current results are concerning. This multicenter and large sample size could be representative of Chinese knowledge workers. Over three years, 37,411 knowledge workers expressed depressive symptoms. Among this population group, approximately one-third (30.8%) of the participants’ DDS scores were below DDS-3. In addition, 89,605 (75.5%) of the knowledge workers still spent more than 2 h of sedentary time outside of work. Together, these reflect serious health risk factors among knowledge workers in China that must be addressed in a targeted manner. This study can be applied to indicate from what aspect to reduce mental workers' depressive symptoms and the intervention programs design of it for Chinese.

## Conclusions

Our study was the first to concentrate on the effect of dietary diversity and sedentary time spent outside of work on depressive symptoms among knowledge workers. We concluded that more sedentary time spent outside of work and lower levels of dietary diversity are risk factors for depressive symptoms. In addition, eating irregularly and overeating are also major risk factors for knowledge workers’ depressive symptoms. Future studies should apply objective indicators to explore the influencing factors contributing to depressive symptoms in this group. Furthermore, programs and interventions should be implemented to improve dietary diversity and reduce the amount of sedentary time spent outside of work among knowledge workers.

## Data Availability

The datasets used and/or analysed during the current study available from the corresponding author on reasonable request.
